# MicroRNA-26b inhibits tumor metastasis by targeting the KPNA2/c-jun pathway in human gastric cancer

**DOI:** 10.18632/oncotarget.8629

**Published:** 2016-04-07

**Authors:** Ming-Ming Tsai, Hsiang-Wei Huang, Chia-Siu Wang, Kam-Fai Lee, Chung-Ying Tsai, Pei-Hsuan Lu, Hsiang-Cheng Chi, Yang-Hsiang Lin, Liang-Mou Kuo, Kwang-Huei Lin

**Affiliations:** ^1^ Department of Nursing, Division of Basic Medical Sciences and Research Center for Industry of Human Ecology, Chang-Gung University of Science and Technology, Taoyuan, 333, Taiwan; ^2^ Department of Biochemistry, College of Medicine, Chang-Gung University, Taoyuan, 333, Taiwan; ^3^ Department of General Surgery, Chang Gung Memorial Hospital at Chia-yi, Chia-yi, 613, Taiwan; ^4^ Department of Pathology, Chang Gung Memorial Hospital, Chia-yi, 613, Taiwan; ^5^ Department of Dermatology, Chang Gung Memorial Hospital, Chang Gung University, Taipei, 10508, Taiwan; ^6^ Liver Research Center, Chang Gung Memorial Hospital, Linkou, Taoyuan, 333, Taiwan

**Keywords:** miR-26b, KPNA2, c-jun, prognosis, gastric cancer

## Abstract

MicroRNAs (miRNA) play an important role in carcinogenesis. Previously, we identified miR-26b as a significantly downregulated miRNA in gastric cancer (GC) tissues (*n* = 106) based on differential quantitative RT-PCR (RT-qPCR) miRNA expression profiles. In the current study, we aimed to clarify the potential role of miR-26b and related target genes in GC progression. Downregulation of miR-26b was associated with advanced tumor-node-metastasis stage (TNM stage) and poor 5-year survival rate. Forced expression of miR-26b led to inhibition of GC cell migration and invasion *in vitro* and lung metastasis formation *in vivo*. Conversely, depletion of miR-26b had stimulatory effects. Additionally, miR-26b affected GC cell behavior through negative regulation of the metastasis promoter, karyopherin alpha 2 (KPNA2). Ectopic expression of miR-26b induced a reduction in KPNA2 protein levels, confirmed by luciferase assay data showing that miR-26b directly binds to the 3′ untranslated regions (UTR) of KPNA2 mRNA. Furthermore, miR-26b and KPNA2 mRNA/protein expression patterns were inversely correlated in GC tissues. Cag A of *Helicobacter pylori* (*Hp*) enhanced miR-26b levels through regulation of the KPNA2/c-jun pathway. Taken together, our data indicate that miR-26b plays an anti-metastatic role and is downregulated in GC tissues via the KPNA2/c-jun pathway. Based on the study findings, we propose that miR-26b overexpression or KPNA2/c-jun suppression may have therapeutic potential in inhibiting GC metastasis.

## INTRODUCTION

Gastric cancer (GC) is one of the most common cancer types, and the second leading cause of cancer-related mortality worldwide [1]. In 2014, GC was reported as the sixth leading cause of cancer mortality in Taiwan [2]. However, the molecular pathogenesis of the disease remains poorly understood. Patients with GC often show poor survival outcomes. The advanced clinical stage system is the only accepted method for predicting prognosis of clinical GC patients with progressive disease, with surgery being the only major therapeutic option. Identification of the pathogenic mechanisms involved in GC and development of useful prognostic biomarkers as well as novel targeted therapeutic strategies is therefore an urgent clinical necessity [3]. Recent studies have revealed the involvement of microRNAs (miRNAs) in the initiation and progression of several cancer types. MiRNAs are dysregulated in many cancers and function either as oncomiRs or tumor suppressors [4, 5]. In a previous study, we assessed differential RT-qPCR miRNAexpression profiles and identified 30 differentially expressed miRNAs in GC tissues, compared with paired normal tissues [6]. Sixteen of these miRNAs were significantly downregulated (< 1.5-fold, *p* < 0.05) and the remaining 14 significantly upregulated (> 1.5-fold, *p* < 0.05) compared to paired normal tissues. Among these, miR-196a/−196b were identified as two of the most significantly upregulated miRNAs in GC tissues associated with promotion of tumor metastasis via downregulation of radixin, supporting their function as prometastatic oncomiRs [6]. Accumulating evidence has shown that miR-26b is downregulated in GC, implying a tumor suppressor role. Zhang *et al.* [7] reported that miR-26b inhibits adipogenic differentiation via suppressing HMGA1 in the ERK1/2 or JNK MAPK and adipogenesis pathways. Tan and colleagues identified a novel estrogen/MYC/miR-26 axis that mediates estrogen-stimulated breast cancer cell proliferation via CHD1, GREB1 and KPNA2 [8, 9]. In the current study, we sought to clarify the potential role and related target genes of miR-26b in GC progression and metastasis.

## RESULTS

### MiR-26b is correlated with poor tumor progression

**Figure 1:**
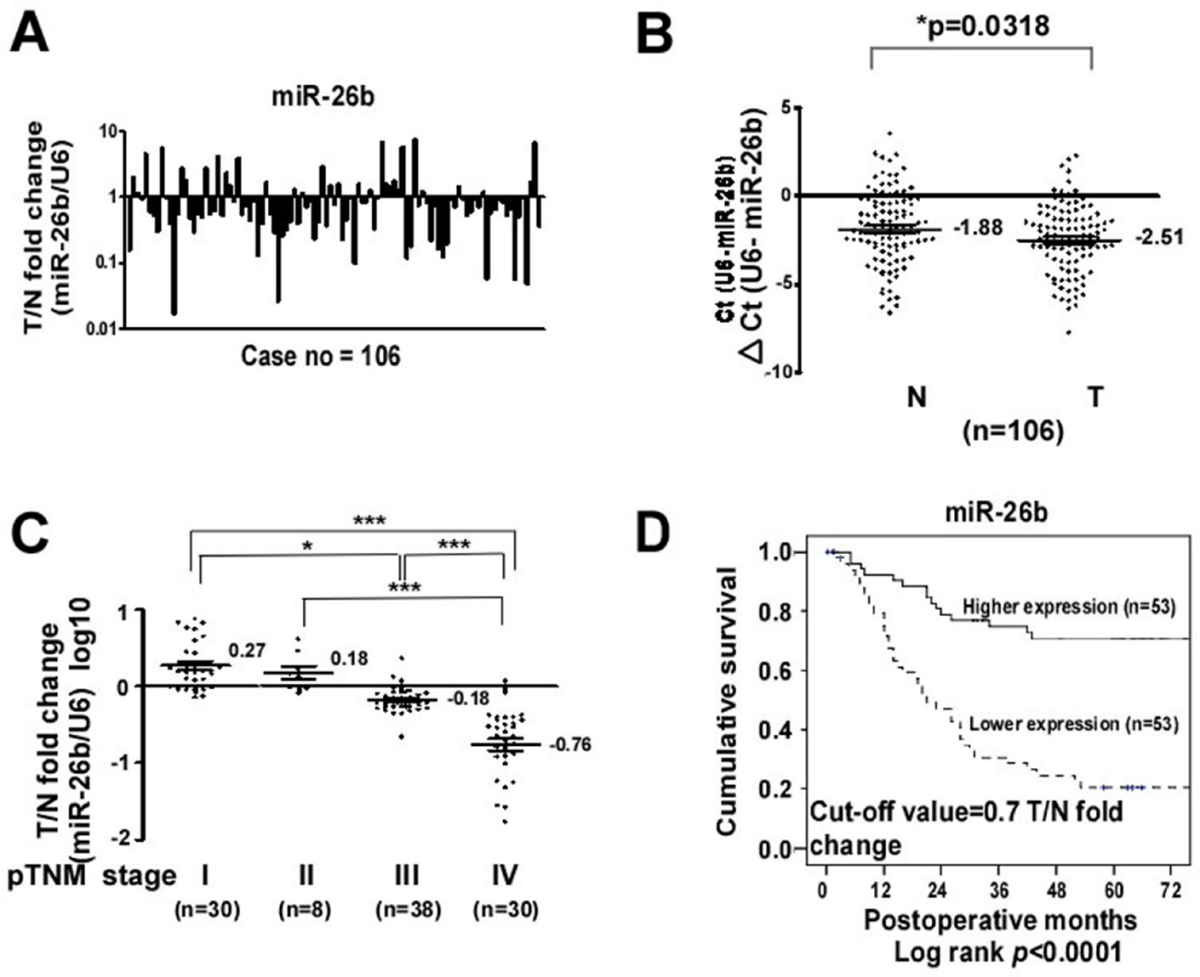
Downregulation of miR-26b in clinical GC tissues is correlated with advanced clinical stage and poor prognosis (**A**) The miR-26b level was downregulated in 67.9% (72 of 106) of GC tumors (T), relative to paired normal tissues (N). (**B**) MiR-26b expression was determined using RT-qPCR and U6 normalization. (**C**) Expression levels of miR-26b at different clinical stages of GC. (**D**) MiR-26b expression vs five-year survival rate in 106 GC tissues. Mann-Whitney *U* test was used for comparison between the two groups. *p < 0.01, **p < 0.05, ***p < 0.001.

In a previous study [6], differential RT-qPCR miRNA expression profile data revealed downregulation of miR-26b in GC tissues relative to paired normal tissues. To further confirm the decrease in miR-26b in GC, RT-qPCR was performed using 106 paired GC and normal tissues. The mean fold change in miR-26b expression in GC tissues was 1.364–fold (range, 0.028-15.589) greater, relative to that in paired normal tissues (Figure 1A). The means of T and N for miR-26b were −1.88 and −2.51, respectively. We observed significant downregulation of miR-26b (1.46-fold) in GC tissues from 75/106 cases (*p* = 0.0318, Mann-Whitney *U* test), consistent with miRNA profiling data (Figure 1B). Considering the definition of downregulation as T/N ratio < 0.5-fold, miR-26b was decreased in 67.92% GC tissues, relative to paired normal tissues. To establish the relationship between miR-26b expression and tumor progression, GC tissues were classified into four subgroups according to clinical stage. Patterns of miR-26b expression displayed a stepwise decrease upon GC progression from the early (I and II) to late (III and IV) stages (*p* < 0.001, Mann-Whitney *U* test, Figure 1C). Our findings collectively indicate that low miR-26b expression is correlated with advanced tumor stage and lymph node metastasis.

### Clinicopathological correlations of miR-26b in GC tissues

The relationships between miR-26b expression patterns and clinicopathological parameters of GC are summarized in Table 1. The characteristics of the study cases are listed (*n* = 106). Mean tumor size (maximum diameter) was 5.6 cm (median, 5.2 cm; range, 0.3–20 cm). Tumors were located in the proximal third of the stomach in 27 (25.5%) cases, middle third in 24 (22.6%) cases, distal third in 50 (47.2%) patients, and the whole stomach in 5 (4.7%) patients. Histological tumor types were intestinal (*n* = 36, 34%) and diffuse (*n* = 70, 66%). As defined by the depth of wall invasion, early GC (T1) was diagnosed in 26 (24.5%), advanced GC (T2) (muscle proper and subserosa) in 13 (12.3%), serosa (T3) in 51 (48.1%), and invasion to adjacent organs (T4) in 16 (15.1%) patients. Lymph node metastasis was diagnosed in 74 (69.8%) patients. During surgery, liver metastasis was identified in three (2.8%), peritoneal seeding in 20 (18.9%), vascular invasion in 26 (24.5%), lymphatic invasion in 61 (57.5%), and perineural invasion in 38 (35.8%) patients. In terms of pathological staging, 30 (28.3%) patients were classified as stage I, 8 (7.5%) as stage II, 38 (35.8%) as stage III and 30 (28.3%) as stage IV. Expression of miR-26b in tumor tissues was not associated with age, gender and liver metastasis. Interestingly, miR-26b levels were closely correlated with location (*p* = 0.002), gross type (*p* < 0.001), histological type (*p* = 0.003), depth of invasion (*p* < 0.001), and serosal invasion (*p* < 0.001). MiR-26b was more significantly downregulated in T3 and T4 groups where the serosal surface of the gastric wall was invaded by cancer, compared to T1 and T2 groups where no invasion was evident (*p* = 0.001), as well as in relation to higher lymph node status (*p* < 0.001), lymph node metastasis (*p* < 0.001), and distant metastasis (*p* < 0.001). Moreover, miR-26b was significantly decreased in patients displaying metastasis to the lymph nodes (*p* < 0.001) and those at more advanced pathologic stages (III and IV) of GC, compared to those in the earlier pathologic stages (I and II) (*p* < 0.001), as well as in patients with peritoneal seeding (*p* < 0.001), vascular invasion (*p* < 0.001), lymphatic invasion (*p* < 0.001) and perineural invasion (*p* < 0.001). Since miR-26b expression was negatively correlated with pathologic features (TNM stage), it is reasonable to hypothesize that loss of miR-26b expression promotes GC metastasis. The prognostic significance of miR-26b downregulation in GC was additionally determined via survival analysis. Univariate analysis with log-rank test disclosed parameters with significant prognostic influence on patient survival (Table 1). Figure 1D illustrates the cumulative survival curves of lower and higher expression groups of miR-26b using the median value (= 0.7) as the cut-off. Median follow-up duration for survivors (*n* = 53) was 83 months (range, 64−137 months). The five-year survival rate of the lower expression group was significantly higher than that of the higher expression group (78.5% vs. 20.8%, log-rank *p* < 0.0001). Multivariate analysis (Cox regression) was performed to determine the independent prognostic potential of miR-26b for GC in relation to the significant clinicopathological parameters in univariate analysis. Histological type (*p* = 0.039, hazards ratio (HR) = 2.263, 95% confidence interval (CI) = 1.042−4.914) and miR-26b (*p* = 0.045, HR = 2.473, 95% CI = 1.02−5.996) emerged as significant independent prognostic biomarkers for GC in a stepwise forward-conditional multivariate regression model.

**Table 1:** Clinicopathological correlations of miR-26b expressions and 5-year survival rate in 106 GC patients

Clinicopathological correlations				Univariate analysis		Multivariate analysis		
Parameters	No.	Mean ± SE^a^	*P*^b^	5-yr S.R.^c^	Log rank *P^d^*	HR	95% CI	*P ^f^*
**Age (yrs)**								
< 65	56	1.07 ± 0.19	**0.418**	41.5	**0.2706**			
≥ 65	50	1.24 ± 0.21		51.9				
**Gender**								
Male	59	1.18 ± 0.20	**0.474**	41.7	**0.3521**			
Female	47	1.12 ± 0.19		52.2				
**Location**								
Upper third	27	1.53 ± 0.38	**0.002**	56.0	**0.0025**			
Middle third	24	1.55 ± 0.24		49.1				
Lower third	50	0.83 ± 0.17		44.3				
Whole	5	0.48 ± 0.17		0.0				
**Gross type**						1.157	0.51−2.626	**0.727**
Localized	44	1.89 ± 0.29	**< 0.001**	74.2	**< 0.0001**			
Infiltrative	62	0.63 ± 0.08		25.4				
**Histological type**						2.263	1.042−4.914	**0.039**
Intestinal	36	1.81 ± 0.35	**0.003**	71.3	**< 0.0001**			
Diffuse	70	0.81 ± 0.10		32.3				
**Depth of invasion (pT)**						0.758	0.244−2.355	**0.632**
T1	26	2.30 ± 0.38	**< 0.001**	91.8	**< 0.0001**			
T2	13	1.96 ± 0.56		59.8				
T3	51	0.63 ± 0.06		29.2				
T4	16	0.30 ± 0.07		13.3				
**Serosal invasion**						2.135	0.818−5.571	**0.121**
No (T1, T2)	39	2.18 ± 0.31	**< 0.001**	81.0	**< 0.0001**			
Yes (T3, T4)	67	0.56 ± 0.05		25.4				
**Lymph node status (pN)**						1.608	0.328−7.878	**0.558**
N0	32	2.15 ± 0.36	**< 0.001**	86.7	**< 0.0001**			
N1	33	1.07 ± 0.18		43.3				
N2	19	0.51 ± 0.07		28.5				
N3	22	0.37 ± 0.10		4.8				
**Lymph node metastasis**								
No (N0)	32	2.15 ± 0.36	**< 0.001**	86.7	**< 0.0001**			
Yes (N1, N2, N3)	74	0.72 ± 0.09		28.2				
**Distant metastasis (pM)**								
No	79	1.46 ± 0.18	**< 0.001**	60.1	**< 0.0001**			
Yes	27	0.24 ± 0.04		4.0				
**Pathological stage (pStage)**						7.771	0.971−62.201	**0.053**
I	30	2.45 ± 0.37	**< 0.001**	92.9	**< 0.0001**			
II	8	1.76 ± 0.41		75.0				
III	38	0.71 ± 0.06		36.1				
IV	30	0.25 ± 0.04		3.6				
**Pathological stage**								
Stages I, II	38	2.30 ± 0.31	**< 0.001**	88.9	**< 0.0001**			
Stages III, IV	68	0.51 ± 0.04		21.9				
**Liver metastasis**								
No	103	1.18 ± 0.14	**0.063**	47.7	**0.1102**			
Yes	3	0.30 ± 0.08		0.0				
**Peritoneal seeding**						1.469	0.552−3.914	**0.441**
No	86	1.36 ± 0.17	**< 0.001**	56.2	**< 0.0001**			
Yes	20	0.26 ± 0.05		0.0				
**Vascular invasion**						1.667	0.893−3.111	**0.108**
No	80	1.40 ± 0.18	**< 0.001**	57.4	**< 0.0001**			
Yes	26	0.39 ± 0.07		8.7				
**Lymphatic invasion**						1.917	0.704−5.218	**0.203**
No	45	2.03 ± 0.28	**< 0.001**	79.2	**< 0.0001**			
Yes	61	0.50 ± 0.05		21.1				
**Perineural invasion**						1.008	0.541−1.877	**0.98**
No	68	1.49 ± 0.20	**< 0.001**	59.5	**< 0.0001**			
Yes	38	0.54 ± 0.08		22.2				
**miR-26b (qRT-PCR)**						2.473	1.02−5.996	**0.045**
< 0.7 (medium) ^e^	53	1.15 ± 0.14		20.4	**< 0.0001**			
≥ 0.7	53			70.9				

a Scores determined by Tagman qRT-PCR in Mean ± SE.

b Mann-Whitney *U* test (for 2 groups) or Kruskal-Wallis test (for > 2 groups).

c Five-year survival rate.

d Log rank test.

e 50th percentile.

f Hazard ratios, 95% CI and *P* values were generated using a Cox proportional hazard analysis.

### MiR-26b inhibits GC cell invasion *in vitro* and metastasis *in vivo*

**Figure 2:**
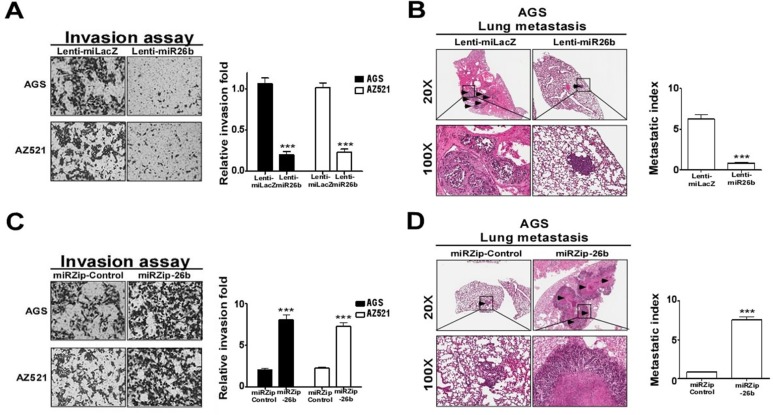
MiR-26b regulates cell invasion Two days after infection (Lenti-miLacZ/Lenti-miR26b or miRZip-control/miRZip-26b virus), blasticidin (8 μg/ml) or puromycin (4 μg/ml) was added, and cells selected for two weeks. The number of cells invading the Matrigel to the lower chamber were determined after (**A**) miR-26b overexpression in AGS and AZ-521 cell lines. (**B**) MiR-26b-overexpressing AGS cells were injected into mouse tail vein (1 × 10^6^ cells). Lung metastases were quantified (right) and images shown (left) *n* = 3. All mice were sacrificed after 10 weeks. (**C**) Invasion activities of miR-26b-depleted AGS and AZ-521 cells. (**D**) Lung metastases from miR-26b-depleted AGS cells injected into mouse tail vein (1 × 10^6^ cells). Lung metastases were quantified (right), and images shown (left) *n* = 3. All mice were sacrificed after 16 weeks. Data are presented as mean values ± SEM. Mann-Whitney *U* test was used for comparison between the two groups.

Since clinicopathological data indicate that miR-26b is closely associated with GC metastasis, we postulated that miR-26b overexpression in GC cells should impede their invasive ability. To examine the association between miR-26b and invasiveness of GC cell lines, miR-26b-overexpressing and -depleted stable AGS and AZ-521 sublines were established. Images of miR-26b-overexpressing and -depleted AGS and AZ-521 cell lines are depicted in Figure S1. Overexpression and suppression of miR-26b were confirmed using RT-qPCR (Figure S1). The effects of aberrant expression of miR-26b on cell migration and invasion activities were assayed using the Transwell method. Notably, miR-26b overexpressing AGS and AZ-521 cells exhibited significantly lower migration rates (5.33- and 5.08-fold, Figure S2A) and invasive abilities (3.61- and 4.55- fold, *p* < 0.001, Mann-Whitney *U* test, Figure 2A), compared to the respective control cell lines. Conversely, miR-26b-depleted cells exhibited markedly higher migration (11.8- and 1.6-fold, *p* < 0.001, Mann-Whitney *U* test, Figure S2B) and invasion rates (2.73- and 2.06-fold, *p* < 0.001, Mann-Whitney *U* test, Figure 2C), relative to control cell lines. To further explore the role of miR-26b in tumor metastasis *in vivo*, SCID mice were transplanted with stable miR-26b-overexpressing or -depleted AGS cells through the lateral tail vein. Histological analysis of lungs of mice confirmed inhibition of lung metastasis nodules by miR-26b. In Figure 2B and 2D, the arrows indicate lung colonies. All lines of SCID mice also developed multiple macroscopic tumor nodules in lung, as observed with H & E staining. The average lung colony formation index was decreased 7.33-fold (*p* < 0.01, Mann-Whitney *U* test, Figure 2B) in miR-26b-overexpressing cells and increased 9.08-fold (*p* < 0.01, Mann-Whitney *U* test, Figure 2D) in miR-26b-depleted cells. Our data strongly indicate a metastasis suppressor role of miR-26b in GC.

### MiR-26b targets KPNA2 to suppress metastasis

**Figure 3:**
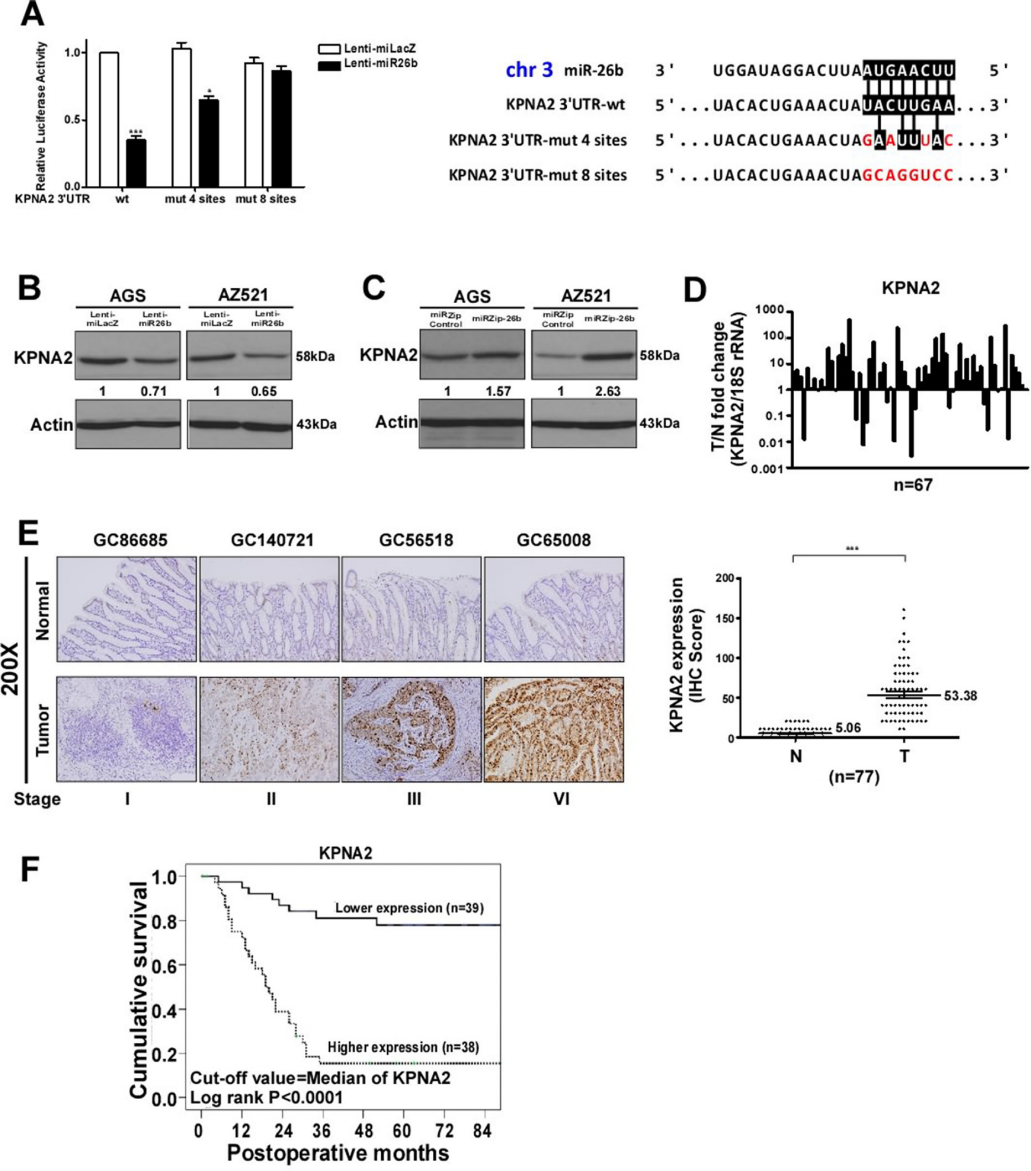
MiR-26b targets KPNA2 (**A**) Wild-type (wt) and mutant (mut) 3′UTRs of KPNA2 were cloned (right) and the luciferase reporter assay performed (left). Immunoblots of KPNA2 gene expression after (**B**) miR-26b overexpression or (**C**) depletion in GC clones. (**D**) KPNA2 mRNA expression was determined via RT-qPCR in T and N. (**E**) IHC staining (left, *N* = 77). KPNA2 expression levels were determined based on IHC scores in T and N (right). Mann-Whitney U test was applied for comparison between the two groups. (**F**) KPNA2 expression vs five-year survival rate in 77 GC tissues. Mann-Whitney *U* test was used for comparison between the two groups. *p < 0.01, **p < 0.05, ***p < 0.001.

To explore the molecular mechanisms underlying the role of miR-26b in GC, TargetScan, a miRBase algorithm, was used and combined with differentially expressed proteins of the GC database from our laboratory isobaric tags for relative and absolute quantification (iTRAQ) (data not shown) to identify the putative protein coding gene targets of miR-26b, particularly those with oncogenic functions (Figure S3A). Consequently, 11 candidate genes (CSTF2, KPNA2, LRPPRC, DRG1, LYN, SFPQ, ALDH5A1, GMFB, SLC12A2, AGPAT5 and DIDO1) were selected (Supplementary Table S1A). Reporter assays were performed with each 3′UTR to screen for genes with activities repressed by miR-26b. The 3′UTR regions of the 11 potential candidate gene sequences were amplified and cloned into pMIR-REPORT Luciferase vector (Supplementary Table S1B). Among these, KPNA2 activity was significantly altered by miR-26b (Figure S3B and S3C). To further ascertain whether KPNA2 is a direct target of miR-26b, the 3′UTR and its corresponding mutant counterparts were fused to the pMIR-REPORT Luciferase vector. Notably, reporter activities of wild-type 3′ UTR, but not mutant 3′UTR, were suppressed by miR-26b. This regulation was dependent on specific sequences, as shown in Figure 3A (left). The results collectively indicate that miR-26b downregulates KPNA2 expression by directly targeting its 3′UTR. In addition, western blot analysis revealed that the KPNA2 level is decreased by 0.71- and 0.65-fold in AGS and AZ-521 cells overexpressing miR-26b, respectively (Figure 3B). Conversely, the KPNA2 level was increased 1.57- and 2.63-fold in AGS and AZ-521 cells depleted of miR-26b, respectively (Figure 3C), confirming regulation of KPNA2 expression by miR-26b in GC. Expression levels of KPNA2 in 67 paired cases were examined to establish its potential clinicopathological associations (Figure 3D). KPNA2 expression was higher in 76.1% of tumor samples, compared to their normal tissue counterparts. KPNA2 protein levels were additionally evaluated in 77 paired cases via IHC (Figure 3E). Mean IHC scores of tumor versus paired normal tissues were 53.38 and 5.06, respectively, with significant differences (*p* < 0.01, Mann-Whitney *U* test). Moreover, 81.8% tumor counterparts displayed higher levels of KPNA2 than normal tissues, suggestive of a role as an oncoprotein in GC. Figure 3F illustrates the cumulative survival curves of lower and higher expression groups of KPNA2 using a median value of 40 as the cut-off. Median follow-up duration of survivors (*n* = 53) was 84 months (range, 64-139 months). The five-year survival rate of the lower expression group was significantly greater than that of the higher expression group (78% vs. 15.4%, log-rank *p* < 0.0001). Relationships between KPNA2 expression and clinicopathological parameters of GC are summarized in Table 2. The characteristics of the study cases are listed (*n* = 77). Tumor size (maximum diameter) was, on average, 5.6 cm (median, 5.2 cm; range, 0.3–20 cm). Tumors were located in the proximal third of the stomach in 17 (22.1%), middle third in 18 (23.4%), distal third in 37 (48.1%), and the whole stomach in 5 (6.5%) cases. Histological tumor types were intestinal (*n* = 22, 28.6%) and diffuse (*n* = 55, 71.4%). As defined based on the depth of wall invasion, early GC (T1) was diagnosed in 16 (20.8%), advanced GC (T2) (muscle proper and subserosa) in 12 (15.6%), serosa (T3) in 37 (48.1%), and invasion to adjacent organs (T4) in 12 (15.6%) cases. Lymph node metastasis was diagnosed in 54 (70.1%) cases. During surgery, liver metastasis was identified in 2 (2.6%), peritoneal seeding in 18 (23.4%), vascular invasion in 20 (26%), lymphatic invasion in 46 (59.7%), and perineural invasion in 28 (36.1%) cases. In terms of pathological staging, 21 (27.3%) cases were classified as stage I, 7 (9%) as stage II, 24 (31.2%) as stage III and 25 (32.5%) as stage IV. Expression of KPNA2 in tumor tissues was not associated with age, gender or liver metastasis. Interestingly, KPNA2 levels were closely correlated with location (*p* = 0.026), gross type (*p* < 0.001), histological type (*p* < 0.001), depth of invasion (*p* < 0.001), and serosal invasion (*p* < 0.001). KPNA2 was more significantly downregulated in T3 and T4 groups whereby the serosal surface of the gastric wall was invaded by cancer, compared to T1 and T2 groups with no evident invasion (*p* < 0.001), as well as in relation to higher lymph node status (*p* < 0.001), lymph node metastasis (*p* < 0.001) and distant metastasis (*p* < 0.001). Moreover, KPNA2 was markedly decreased in patients displaying metastasis to the lymph node (*p* < 0.001) and more advanced pathologic stages (III and IV) of GC, compared to earlier pathologic stages (I and II) (*p* < 0.001), as well as those with peritoneal seeding (*p* < 0.001), vascular invasion (*p* < 0.001), lymphatic invasion (*p* < 0.001) and perineural invasion (*p* < 0.001). Since KPNA2 expression is negatively correlated with pathologic features (TNM stage), it is reasonable to hypothesize that loss of KPNA2 promotes GC metastasis. The prognostic significance of KPNA2 upregulation in GC was further determined via survival analysis.

**Table 2:** Clinical characteriscs of 77 GC patients in two groups according to KPNA2 expression

Parameters	No.	Mean ± SE^a^	*P*^b^	Parameters	No.	Mean ± SE^a^	*P*^b^
**Age (years)**				**Lymph node metastasis**			
< 65	36	50.8 ± 5.8	**0.586**	No (N0)	23	27.0 ± 7.6	**< 0.001**
≥ 65	41	55.6 ± 5.9		Yes(N1,N2,N3)	54	64.6 ± 4.1	
**Gender**				**Distant metastasis(pM)**			
Male	41	50.3 ± 5.9	**0.411**	No	56	38.0 ± 3.4	**< 0.001**
Female	36	56.1 ± 5.9		Yes	21	94.3 ± 6.5	
**Location**				**Pathological stage(pStage)**			
Upper third	17	51.8 ± 10.1	**0.026**	Stages I	21	15.2 ± 2.5	**< 0.001**
Middle third	18	38.3 ± 6.5		Stages II	7	30.0 ± 3.1	
Lower third	37	55.7 ± 5.6		Stages III	24	54.2 ± 3.7	
Whole	5	96.0 ± 15.0		Stages IV	25	91.2 ± 5.8	
**Gross type**				**Pathological stage**			
Localized	29	30.0 ± 6.0	**< 0.001**	Stages I, II	28	18.9 ± 2.3	**< 0.001**
Infiltrative	48	67.5 ± 4.5		Stages III, IV	49	73.1 ± 4.4	
**Histological type**				**Liver metastasis**			
Intestinal	22	33.2 ± 7.5	**< 0.001**	No	75	52.3 ± 4.1	**0.303**
Diffuse	55	61.5 ± 4.6		Yes	2	95.0 ± 55.0	
**Depth of invasion(pT)**				**Peritoneal seeding**			
T1	16	16.3 ± 2.7	**< 0.001**	No	59	43.2 ± 4.3	**< 0.001**
T2	12	23.3 ± 4.0		Yes	18	86.7 ± 6.6	
T3	37	67.8 ± 5.1		**Vascular invasion**			
T4	12	88.3 ± 7.2		No	57	41.8 ± 3.8	**< 0.001**
**Serosal invasion**				Yes	20	86.5 ± 8.1	
No (T1,T2)	28	22.1 ± 4.0	**< 0.001**	**Lymphatic invasion**			
Yes(T3,T4)	49	71.2 ± 4.4		No	31	30.0 ± 5.8	**< 0.001**
**Lymph node status(pN)**				Yes	46	69.1 ± 4.5	
N0	23	27.0 ± 7.6	**< 0.001**	**Perineural invasion**			
N1	25	49.6 ± 5.0		No	49	40.4 ± 4.9	**< 0.001**
N2	12	65.0 ± 5.7		Yes	28	76.1 ± 5.5	
N3	17	86.5 ± 7.7		**KPNA2 (IHC score)**			
				< 40 (median)	38		
				≥ 40	39		

a KPNA2 scores detected by IHC method in mean ± standard error (SE). IHC; immunohistochemistry.

b *P* values are analyzed by Pearson's chi-square test or Fisher's exact test.

### Validation of KPNA2 as an oncoprotein in GC

KPNA2 is closely associated with GC metastasis, and identified as a putative pro-metastatic gene in several studies [10–12]. To further explore its function, KPNA2-overexpressing and depleted AGS and AZ-521 cell lines were established. KPNA2 expression was decreased by 3.45- and 3.85-fold or increased 3.16- and 3.7-fold, respectively, as confirmed with western blot (Figure S5A). AGS and AZ-521 cells treated with shKPNA2 exhibited significantly decreased migration (4.35- and 3-fold, respectively) and invasion (3.45- and 2.71-fold, respectively) (*p* < 0.01, Mann-Whitney *U* test), relative to those transfected with control vector (Figure S5B). Conversely, KPNA2 overexpression led to significantly increased migration and invasion (Figure S5C). Our data collectively support a pro-migratory and invasion-stimulating role of KPNA2. To further explore the function of KPNA2 *in vivo*, the gene was either depleted or overexpressed in AGS cell lines. KPNA2-depleted cells exhibited a significant (*p* < 0.01, Mann-Whitney *U* test) decrease in metastatic ability *in vivo* (6.52-fold), relative to those transfected with control vector (Figure S5D). Conversely, overexpression of KPNA2 led to markedly increased *in vivo* metastatic ability (5.07- fold) (Figure S5E). The data collectively support a pro-metastatic function of KPNA2.

### KPNA2 is involved in miR-26b-mediated suppression of cell migration and invasion

**Figure 4:**
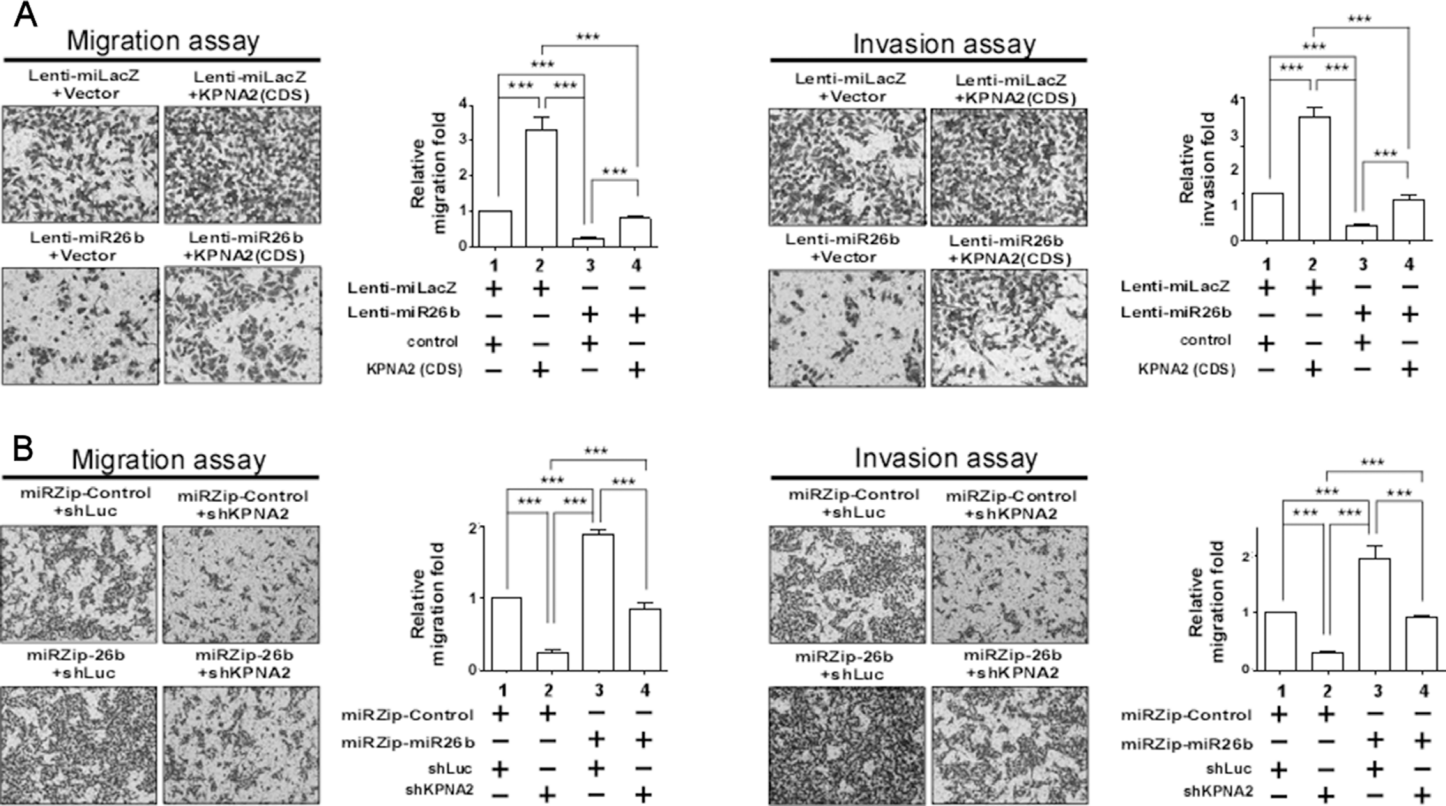
Alterations in KPNA2 expression influence miR-26b-mediated migration and invasion Migration and invasion assays using stable AGS and AZ-521 cells (**A**) GC clones stably overexpressing miR-26b were transfected with KPNA2 or control plasmid. (**B**) MiR-26b-depleted clones were transfected with shKPNA2 or control plasmid. Data are presented as mean values ± SEM. Mann-Whitney *U* test was used for comparisons between the two groups.

To further confirm the association between miR-26b and KPNA2 in GC metastasis, rescue experiments were performed. Re-expression of KPNA2 in a miR-26b-overexpressing line or its knockdown in a miR-26b-depleted line was established (Figure S4). MiR-26b overexpression led to significant inhibition in cell migration and invasion, which was partially rescued upon re-expression of KPNA2 (Figure 4A). Conversely, the migration and invasion abilities of AGS were partially suppressed by shKPNA2 in miR-26b-depleted line (Figure 4B).

### Downregulation of miR-26b is inversely correlated with KPNA2 expression through CagA and c-jun

**Figure 5:**
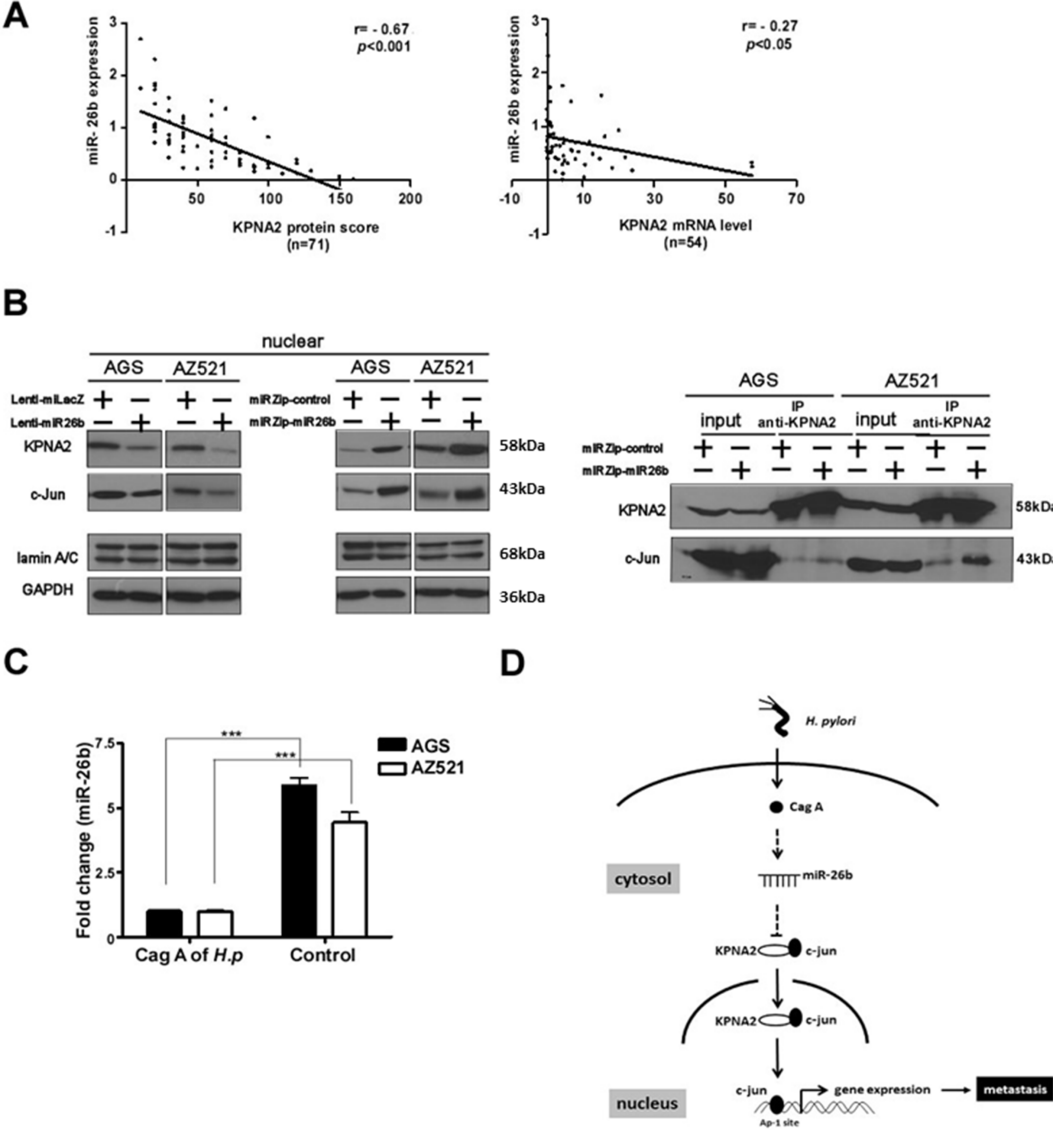
Downregulation of KPNA2 is inversely correlated with miR-26b expression in GC tissues The relationship between miR-26b and KPNA2 protein levels was assessed using (**A**) RT-qPCR and IHC analyses. (**B**) Cag A stimulates miR-26b expression (**C**) Alterations in miR-26b expression influence KPNA2 and c-Jun (left panel) CoIP (right panel) (**D**) Diagram of miR-26b signaling. Data are presented as means ± SEMs. Mann-Whitney *U* test was applied for comparison between the two groups.

An inverse association between KPNA2 protein or mRNA and miR-26b expression was observed in clinical specimens (*n* = 71; *r* = − 0.67, *p* < 0.001 or *n* = 54; *r* = − 0.27, *p* < 0.05, Pearson correlation, Figure 5A). To establish the mechanisms underlying suppression of miR-26b in GC, we explored the potential regulatory factors of miR-26b. To date, a number of risk factors for gastric cancer, including *Helicobacter pylori* (*Hp*) infection, chromosomal instability and genetic alterations, have been identified in GC [13–15]. Therefore, we attempted to determine the factors accounting for lower expression of miR-26b. Based on previous findings, *Helicobacter pylori* (*Hp*) virulence factor CagA (cytotoxin-associated gene A) associated with GC was selected for testing [16]. RT-qPCR analysis revealed 5.87 and 4.43-fold downregulation of miR-26b in CagA-transformed AGS/AZ521 cells (Figure 5C), respectively, indicating that miR-26b is suppressed by CagA. However, the current experiments do not rule out the involvement of other potential factors. Recently, several groups have identified KPNA2 as a nuclear transport receptor protein [17, 18]. Accordingly, we tested the interactions of KPNA2 protein with other factors, including c-jun, c-myc, p53 and E2F1. Notably, c-myc, p53 and E2F1 levels in cell lines stably expressing miR-26b remained unchanged on a western blot (data not shown), while c-jun was significantly suppressed by miR-26b. The effect of miR-26b on c-jun was further examined in the two cell lines. Simultaneous downregulation of KPNA2 and c-Jun proteins was observed in miR-26b-overexpressing cells, suggesting a critical axis of miR-26b-targeted KPNA2/c-jun for GC metastasis and vice versa (Figure 5B, left). In addition, KPNA2 co-immunoprecipitated (CoIP) with c-jun (Figure 5B, right). Thus, miR-26b may be a useful independent prognostic tumor marker to predict survival and GC metastasis. Augmentation of miR-26b signaling or function presents a potential therapeutic strategy for inhibition of GC metastasis (Figure 5D).

## DISCUSSION

MiR-26b is downregulated in various tumor types [19–25], suggesting an important role in tumorigenesis and tumor progression. Previous studies have reported that miR-26b has anti-metastasis (PTEN, EphA2, LARP1, CTGF, PFKFB3, Smad1, TNKS1BP1, CPSF7, COL12A1, Nampt, COX-2 pathway targets), anti-proliferative (CDK8, PTGS2, pRb, CHD1, KPNA2, PTEN-AKT pathway targets), anti-survival (Nampt), anti-epithelial-mesenchymal transition (USP9X), pro-chemosensitivity (NF-κB signaling, TAK1 and TAB3), glycolytic metabolism (PFKFB3) and anti-apoptosis (Smad4, SLC7A11) functions [7, 26–39].

Li *et al.* [45] reported roles of miR-26b in hepatocellular carcinoma (HCC) cell proliferation, migration, and invasion, and confirmed that EphA2 is a direct target. Additionally, miR-26b was shown to downregulate c-Myc and Cyclin D1 expression [35]. The group of Zhu showed that miR-221 and miR-26b enhance mesenchymal stem cell (MSC) migration towards hepatocyte growth factor (HGF) through activation of PI3K/Akt signaling and targeting phosphatase and tensin homolog deleted on chromosome ten (PTEN) *in vitro* [26]. Downregulation of miR-26b modulates chemoresistance and migration through association with PTEN in non-small cell lung cancer cells and human carcinoma tissues [36]. La-related protein 1 (LARP1) was identified as a regulatory target of miR-26a/b inhibiting prostate cancer (PCa) cell invasion by Kato *et al.* [37]. Duan and co-workers found that downregulation of miR-26b in osteosarcoma elevates the levels of CTGF and Smad1, facilitating metastasis in osteosarcoma (os) [27]. Reduced miR-26b expression promoted breast fibroblast migration and invasion. Three novel miR-26b targets were identified (TNKS1BP1, CPSF7, COL12A1), and the expression of each in cancer stroma shown to be significantly associated with breast cancer recurrence [38]. Another study reported that miR-26b acts as a tumor suppressor in glioma and directly regulates EphA2 expression [39].

The role of miR-26b in GC progression remains to be established. In the present study, we identified miR-26b as a novel anti-metastatic miRNA in GC significantly associated with clinically advanced stages and lymph node metastasis. Clinicopathological data indicate that lower expression of miR-26b is associated with GC progression. Additionally, lymph node metastasis is an initial step of GC progression that is associated with clinical stage, prognosis, and survival of GC patients [40]. Recently, miRNAs linked to lymph node metastasis of GC, such as miR-218, miR-146a, miR-429, and miR-370, have been identified [41–44]. These findings may provide new insights into ways to design improved therapeutic strategies for GC patients with metastasis. Data from the current study revealed that downregulation of miR-26b enhances GC cell migration and invasion *in vitro* and metastasis *in vivo*. KPNA2 was further identified as a direct target of miR-26b. In terms of the underlying mechanism, our experiments showed that CagA of *Hp* stimulates miR-26b downregulation, although further studies are essential to clarify any potential additional factors involved. Notably, miR-26b suppressed KPNA2, both at the mRNA and protein level. KPNA2 (also designated importin α-1 or RAG cohort 1) belongs to a member of the karyopherin α family and plays a key role in the nuclear import of cargo proteins. KPNA2 is implicated in a multitude of cellular processes, including differentiation, transcriptional regulation, immune response, viral infection, cellular maintenance and carcinogenesis. Moreover, aberrant KPNA2 expression has been observed in several cancer types, suggesting a potential role in tumorigenesis [45]. Several studies have shown that KPNA2 acts as an oncogene [10–12], while others report a tumor suppressor role [46]. Here, we demonstrated that KPNA2 is upregulated in GC tissues and increases cell mobility, supporting a pro-metastatic function. Kim *et al.* [47] identified the transcription factor, Sp1, as a common regulator of NCT (nucleocytoplasmic trafficking) genes, including various nucleoporins, importins, exportins, and Ran GTPase cycle-related genes. Our western blot and CoIP analyses additionally led to the identification of c-Jun as a downstream target of KPNA2. c-Jun is a proto-oncogene belonging to a nuclear phosphoprotein family, which heterodimerizes with c-fos to form the transcription factor complex, AP-1. Overexpression of c-fos promotes cell invasion and migration [48]. Furthermore, the public NCBI web GEO profile documents higher KPNA2 expression in 22 primary advanced GC tissues, compared to 8 normal controls (mean = 119.9, 35.3). Similar results have been obtained from the Public IHC database (http://www.proteinatlas.org/) (*N* = 9, T = 22). However, the precise roles of KPNA2 in cancer cells require further investigation.

In summary, our results show that miR-26b, an important metastatic suppressor miRNA, is downregulated in relation to lymph node metastasis progression in GC. Moreover, miR-26b and histological type, a pathological parameter, have independent prognostic value for 5-year cumulative survival, supporting a potential role of metastasis-suppressing miR-26b as a novel prognostic biomarker. Ectopically expressed miR-26b inhibits GC cell invasion and metastasis through direct targeting and negative regulation of KPNA2, and its frequent downregulation is suggested to be part of the mechanism underlying metastasis and progression. Therefore, overexpression of miR-26b or suppression of KPNA2 may have therapeutic potential in GC patients with metastasis. Further research is warranted to establish the potential of miR-26b as a prognostic and therapeutic agent.

## MATERIALS AND METHODS

### Patients

All 106 patients (59 males, 47 females; age: 63.5 years, range, 28–84 years) diagnosed pathologically with GC at the Chang Gung Memorial Hospital (CGMH) from 2000 to 2006 were enrolled in the study. Individual patients were subjected to gastric resection (74 partial gastrectomy and 32 total gastrectomy) as described previously [6]. This study was approved by the Institutional Review Board (IRB No. 102-3017C).

### Clinicopathology

Resected GC specimens were examined pathologically using criteria of the 6th edition of the American Joint Committee on Cancer (AJCC) (TNM) classification system and the Japanese General Rules for GC study [49]. Patient parameters included age, gender, location, gross type, histological type, depth of invasion, serosal invasion, lymph node status, lymph node metastasis, distant metastasis, pathological stage, liver metastasis, peritoneal seeding, vascular invasion, lymphatic invasion, and perineural invasion. After release, all patients had periodic follow-up visits at the outpatient department of CGMH until death or beginning of preparation of this study.

### GC tissues

Fresh GC and paired normal tissues were collected immediately following gastric resection. Specimens were immediately snap-frozen in individual vials using liquid nitrogen. Frozen specimens were stored at −70°C in a tumor bank until use.

### RNA extraction

Total RNA were extracted using TRIzol^®^ Reagent (Invitrogen, Carlsbad, CA). The concentrations of all RNA samples were quantified using NanoDrop 1000 (Nanodrop Technologies Inc., Wilmington, DE).

### RT-qPCR

We assessed miR-26b and KPNA2 mRNA expression patterns as described earlier in the same patient groups [6]. Briefly, to quantify miRNA transcripts, total RNA was extracted from cells using the TRIzol solution kit (Life Technology Inc., Carlsbad, CA, USA). RT-qPCR products were detected using SYBR Green, as described previously, with U6 as the internal control for miRNA [6, 50, 51]. Fluorescence emitted by SYBR green was examined using the ABI PRISM 7500 sequence detection system (Applied Biosystems, Werrington, UK). Sequences of primers used for RT-qPCR were as follows: KPNA2 (forward primer, 5′–TGCTACTTCTCCGCTGCAGG–3′, and reverse primer, 5′–CTGGCAGCTTGAGTAGCTTG–3′); human 18s rRNA (forward primer, 5′–CGAGCCGCCTGGATACC–3′, and reverse primer, 5′–CCTCAGTT CCGAAAACCAACAA–3′).

### Cell culture

Human GC lines (AGS and AZ-521) were obtained from American Type Culture Collection (ATCC) and routinely grown in Roswell Park Memorial Institute medium (RPMI) 1640 (Invitrogen) supplemented with 10% (v/v) fetal bovine serum (FBS) (Invitrogen), 100 IU/ml penicillin G and 100 mg/ml streptomycin sulfate (Sigma-Aldrich). Cells were cultured at 37^°^C in a humidified atmosphere containing 5% CO_2_.

### Establishing a miR-26b-overexpression cell line

Cells were transfected with BLOCK-iT Pol ll miR RNAi Expression vectors (Invitrogen), according to a previously reported protocol [6, 51].

### Establishing a miR-26b-depleted cell line

Cells were transfected with the miRZip-26b lentivector (SBI System, BioScience, Mountain View, CA) or negative control (miRZip lentivector), according to a previous protocol [6, 51].

### *In vivo* metastatic assays

Male severe combined immunodeficiency (SCID, C.B17/Icr-scid) mice 4-6 weeks of age were injected with 1 × 10^6^ cells/200 μl PBS via the tail vein and sacrificed after 8 weeks. All animal assays were performed in accordance with the guidelines of U.S. National Institutes of Health and the Chang-Gung Institutional Animal Care and Use Committee Guide for Care and Use of Laboratory Animals. This study was conducted under the approval of Chang-Gung Institutional Animal Care and Use Committee (IACUC Approval No. CGU13-127). Lung metastasis was quantified by counting the total tissue area per lung section (A1) and metastasis present in the same area (A2). The metastatic index was calculated based on the A2/A1 ratio.

### Luciferase assay

All transient transfections were conducted using TurboFect Reagent (Invitrogen) and pMIR-report plasmids (Ambion, Foster City, CA) as reporter constructs. Cells were harvested 48 h after transfection in lysis buffer and subsequently assayed for luciferase activity (Luciferase Assay System, Promega, WI) using β-galactosidase as the internal control. Cleavage stimulation factor subunit 2 (CSTF2), karyopherin alpha 2 (KPNA2), leucine-rich pentatricopeptide repeat containing (LRPPRC), developmentally regulated GTP binding protein 1 (DRG1), v-yes-1 Yamaguchi sarcoma viral related oncogene homolog (LYN), splicing factor proline/glutamine-rich (SFPQ), aldehyde dehydrogenase 5 family, member A1 (ALDH5A1), glia maturation factor beta (GMFB), solute carrier family 12, member 2 (SLC12A2), 1-acylglycerol-3-phosphate O-acyltransferase 5 (AGPAT5) and Death inducer-obliterator 1 (DIDO1) were assayed. Luciferase reporter genes driven by 3′UTRs of computationally predicted (wt-3′UTR) and mutated KPNA2 (mt-3′UTR) were generated via PCR amplification from human genomic DNA and subsequently cloned between *Mlu* I and *Spe* I sites within the 3′UTR of pMIR-report plasmids. The primer sequences for miR-26b binding sites of 11 putative target genes are provided (Supplementary Table S1).

### Immunohistochemistry (IHC)

Paraffin-embedded tissues (5 μm thickness) were prepared for different GC samples, and IHC performed to detect KPNA2 (Epitomics, Burlingame, CA; dilution 1:150), as described previously [52].

### *In vitro* metastatic assays

To examine the effects of miR-26b and KPNA2 overexpression or depletion on the migratory and invasive activities of GC cell lines, the rapid *in vitro* Transwell assay (Becton-Dickinson, Franklin Lakes, NJ) with 8 mm membrane pore size chamber inserts non-matrigel-coated (migration assay) or matrigel-coated (invasion assay) was performed for quantifying potential tumor cell migration or invasion [53]. Migratory or invasive cells were counted using Image J software.

### Western blot analysis

Total cell lysates from GC and paired normal tissues or cell lines were prepared, and protein concentrations determined using a previously described method [52]. The intensities of immunoreactive bands were quantified using Image Gauge software (Fuji Film, Tokyo, Japan) on a densitometer.

### Gene Ontology database

We used TargetScan, a miRBase algorithm, combined with differentially expressed proteins from the GC database as well as isobaric tags for relative and absolute quantification (iTRAQ) datasets from our laboratory to identify putative protein- coding gene targets of miR-26b. A list of genes related to oncogenic function was compiled based on publicly available databases (Gene Ontology, http://www.geneontology.org) and recent literature. Consequently, 11 candidate genes were selected (the gene list and corresponding Gene Ontology Database are presented in Supplementary Table S1).

### Overexpression/depletion of KPNA2 in GC cell lines

KPNA2 cDNA (no 3′UTR) was amplified using RT-PCR and cloned into the pcDNA3 vector. Transfection of pcDNA3-KPNA2 was performed using TurboFect Reagent (Invitrogen). After 24 h of incubation, cells were transferred to G418 medium for selection. KPNA2 overexpression was confirmed with western blot analysis. Cells transfected with the pcDNA3 vector served as the control. Alternatively, KPNA2 was depleted using specific shRNA, with the aim of determining its function. Clones of shRNA targeting KPNA2 (TRCN0000065309, TRCN00000286475) were purchased from the National RNA Interference Core Facility (Academia Sinica, Taiwan). Transfection of shRNA targeting endogenous KPNA2 genes was performed using TurboFect Reagent (Invitrogen). After 24 h of incubation, cells were transferred to medium containing puromycin for selection. Following 2 weeks of selection, specific suppression of the KPNA2 gene was confirmed with western blot analysis.

### Statistical analysis

The Mann-Whitney *U*-test or Fisher's exact test was used for between-group comparisons where appropriate, and correlation between the results obtained with the two different analyses determined with Spearman's test. Follow-up studies of patients were performed until the time of writing or patient death. Cancer-specific survival outcomes were evaluated by applying the Kaplan-Meier method for all patients, except those who died from surgical complications. The log-rank test was used to compare the prognostic significance of individual variables on survival. Cox's proportional hazards model was applied in multivariate analysis to identify independent predictors of survival. *P*-values < 0.05 were considered statistically significant.

## SUPPLEMENTARY MATERIALS FIGURES AND TABLE



## Abbreviations

AGPAT5: 1-acylglycerol-3-phosphate O-acyltransferase 5
ALDH5A1: aldehyde dehydrogenase 5 family, member A1
Cag A: cytotoxin-associated gene A
CDS: coding sequence
CGMH: Chang Gung Memorial Hospital
CI: confidence interval
CoIP: co-immunoprecipitate
CSTF2: cleavage stimulation factor subunit 2
DIDO1: death inducer-obliterator 1
DRG1: developmentally regulated GTP binding protein 1
GC: gastric cancer
GMFB: glia maturation factor beta
Hp: *Helicobacter pylori*
HGF: hepatocyte growth factor
HR: hzards ratio
IHC: immunohistochemistry
iTRAQ: isobaric tags for relative and absolute quantitation
KPNA2: karyopherin alpha 2
LARP1: La-related protein 1
LRPPRC: leucine-rich pentatricopeptide repeat containing
LYN: v-yes-1 Yamaguchi sarcoma viral related oncogene homolog
MiRNAs: MicroRNAs
MSCs: mesenchymal stem cells
NCT: nucleocytoplasmic trafficking
NSCLC: non-small cell lung cancer
PCa: prostate cancer
PTEN: phosphatase and tensin homologue deleted on chromosome ten
RT-qPCR: real-time reverse transcription quantitative polymerase chain reaction
SCID: severe combined immunodeficiency
SFPQ: splicing factor proline/glutamine-rich
SLC12A2: solute carrier family 12, member 2
TNM stage: tumor-node-metastasis stage
UTR: untranslated regions
