# Supplementary material for: MicroRNA-26b inhibits tumor metastasis by targeting the KPNA2/c-jun pathway in human gastric cancer
Source: Oncotarget. 2016 Apr 7;7(26):39511–26. doi: 10.18632/oncotarget.8629 (PMC5129949; doi:10.18632/oncotarget.8629)
Supplement: Supplementary file 1 [file oncotarget-07-39511-s001.pdf]

MicroRNA-26b inhibits tumor metastasis by targeting the KPNA2/c-jun pathway in human gastric cancer

Supplementary Materials

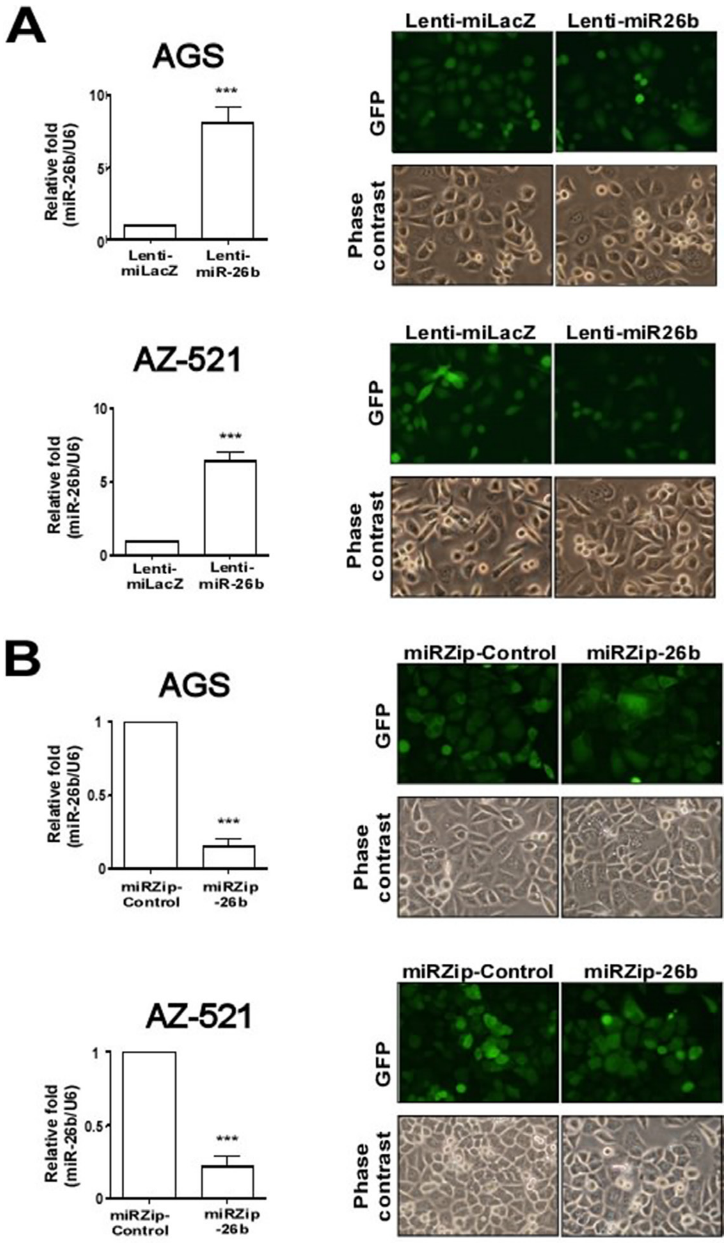

Supplementary Figure S1: Overexpression or depletion of miR-26b in GC cell lines. Two days after lenti-miLacZ or lenti-miR-26b virus infection in AGS and AZ-521 cell lines, blasticidin (8 μg/ml) was added to the medium and stable clones selected. Images of stable cell lines were photographed using phase contrast and GFP staining. RT-qPCR was employed to determine induction fold of miR-26b. (**B**) Two days after miRZip-control and miRZip-26b virus infection into AGS and AZ-521 cell lines, puromycin (4 μg/ml) was added to the medium and stable clones selected. Images of stable cell lines were obtained using phase contrast and GFP staining. RT-qPCR was employed to determine suppression fold of miR-26b. Mann-Whitney *U* test was applied for comparison between the two groups. ***$p < 0.001$.

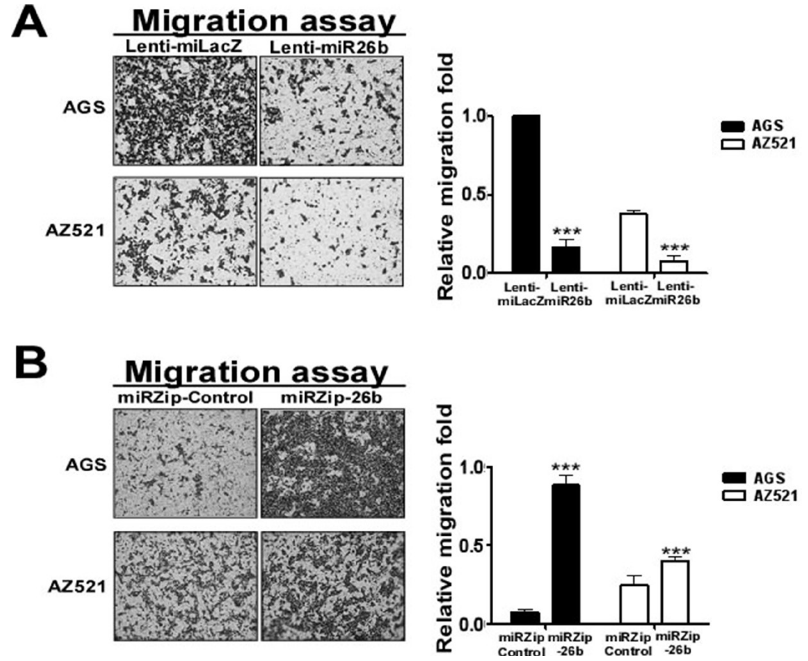

Supplementary Figure S2: MiR-26b inhibits cell migration. MiR-26b overexpression or depletion in AGS and AZ-521 cell lines. Assay of migration activities. The number of cells invading the Matrigel to the lower chamber was determined under conditions of overexpression (**A**) or **(B)** depletion of miR-26b in cell lines. Data are presented as mean values ± SEM. Mann-Whitney *U* test was applied for comparison between the two groups.

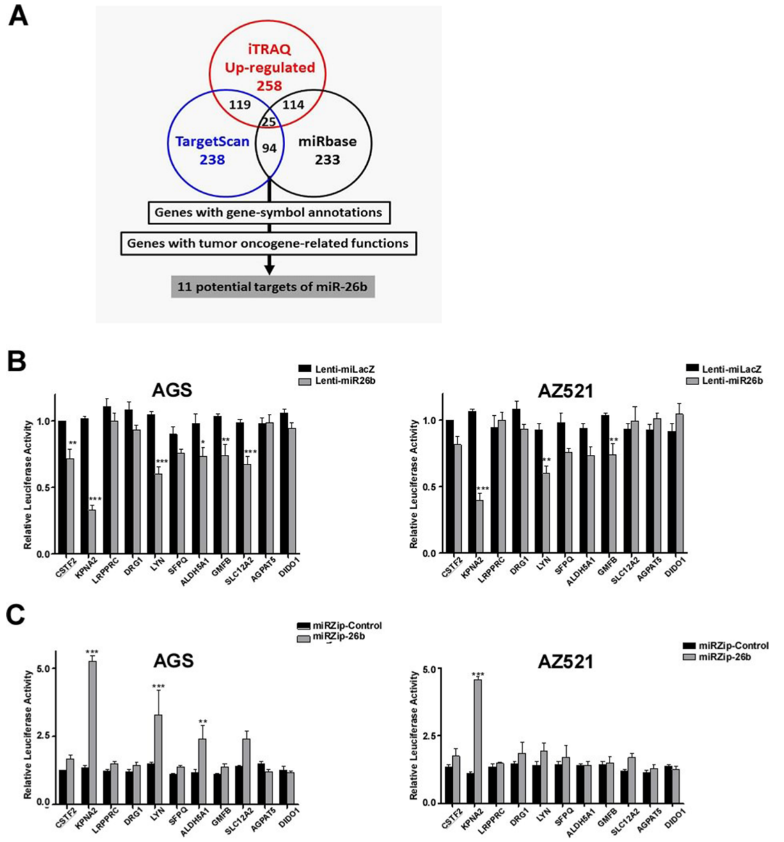

Supplementary Figure S3: KPNA2 is a direct target of miR-26b. (**A**) iTRAQ, TargetScan and miRBase were used for prediction of potential targets of miR-26b. In total, 11 genes were selected as potential targets. (**B**) The luciferase reporter activity assay was performed on 60 bp 3′UTRs from the potential targets using AGS or AZ521 cells stably overexpressing miR-26b. Only KPNA2 displayed > 60% inhibition. (**C**) Conversely, the luciferase reporter activity assay was performed after depletion of miR-26b in the two GC cell lines. Data are presented as mean values ± SEM. Mann-Whitney *U* test was applied for comparison between the two groups.

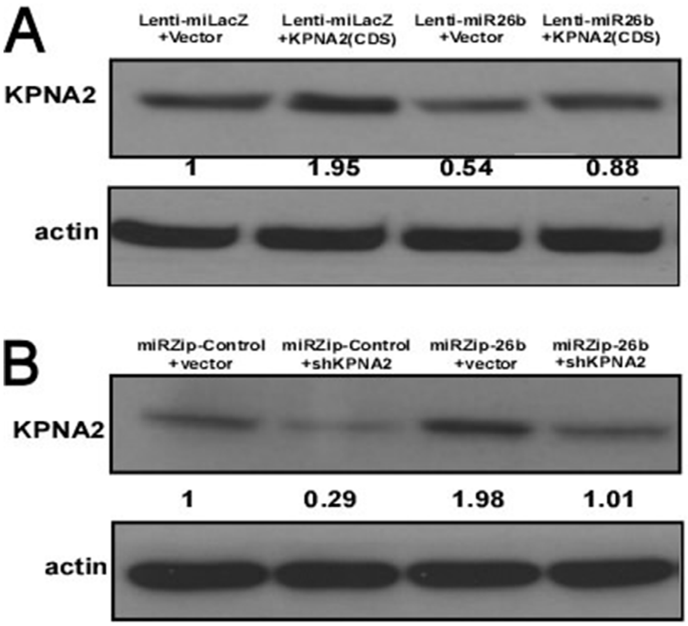

Supplementary Figure S4: MiR-26b-mediated knockdown or overexpression of KPNA2 by. (**A**) re-expression of KPNA2 and (**B**) depletion of KPNA2 with shRNA was confirmed using western blot in AGS cells.

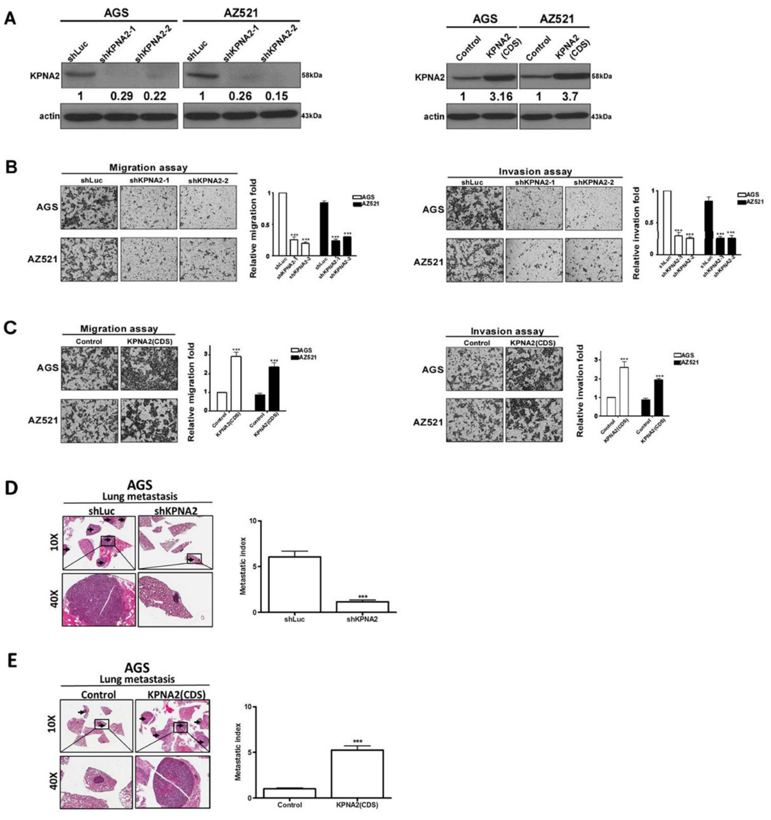

Supplementary Figure S5: Oncogenic role of KPNA2. AGS and AZ-521 cell lines overexpressing or depleted of the *KPNA2* gene were established and selected with puromycin (4 μg/ml) for two weeks. Migration and invasion assays of AGS cells were performed after (**A**) depletion and overexpression of KPNA2 with shRNA was confirmed using western blot in AGS and AZ-521 cells. (**B**) *KPNA2* depletion (shLuc or shKPNA2-1/-2) or (**C**) overexpression (Control or pKPNA2). Results were quantified for *KPNA2* depletion and overexpression. KPNA2-depleted or -overexpressing AGS stable lines were established. Images of lung foci in (**D**) KPNA2 knockdown (shLuc or shKPNA2 plasmid) or (**E**) overexpression (control or pcDNA3-KPNA2 (CDS) plasmid) mice. Stable KPNA2-expressing clones were injected into the mouse tail vein (1×10^6 cells). All mice were sacrificed after 15 weeks. Images of lung metastases were photographed, and the results quantified ($n = 3$). Data are presented as mean values ± SEM. The Mann-Whitney *U* test was used for comparisons between the two groups. *$p < 0.01$, **$p < 0.05$, ***$p < 0.001$.

Supplementary Table S1: (A) Prediction and annotations of potential miR-26b target genes (B) PCR primers for miR-26b binding sites in the 11 putative target genes

(A)

No	HGNC symbol	Description	Function
1	CSTF2	Cleavage stimulation factor subunit 2	Involved in the 3′ end cleavage and polyadenylation of pre-mRNAs
2	KPNA2	Karyopherin alpha 2	It interacts with the nlss of DNA helicase Q1 and SV40 T antigen and may be involved in the nuclear transport of proteins
3	LRPPRC	Leucine-rich pentatricopeptide repeat containing	It may play a role in cytoskeletal organization, vesicular transport, or in transcriptional regulation of both nuclear and mitochondrial genes
4	DRG1	Developmentally regulated GTP binding protein 1	Involved in development process
5	LYN	LYN proto-oncogene, Src family tyrosine kinase	Involved in the regulation of mast cell degranulation, and erythroid differentiation
6	SFPQ	Splicing factor proline/glutamine-rich	Pre-mRNA splicing factor
7	ALDH5A1	Aldehyde dehydrogenase 5 family, member A1	Involved in the metabolism of the neurotransmitter
8	GMFB	Glia maturation factor beta	Involved in glia maturation
9	SLC12A2	Solute carrier family 12, member 2	Mediates sodium and chloride transport and eabsorp-tion
10	AGPAT5	1-acylglycerol-3-phosphate O-acyltransferase 5	Converts lysophosphatidic acid to phosphatidic acid
11	DIDO1	Death inducer-obliterator 1	Apoptotic signal

(B)

No	HGNC symbol	3′UTR Sequence
1	CSTF2	Forward 5′-ATTATACTAGTTAGGTTTTCAAAAATACCTG - 3′<br>Reverse 5′-ATTATAAGCTTGCAGTGTACTAAAATTGTGT - 3′
2	KPNA2	Forward 5′-ATTATACTAGTATCATGTAGCTGAGACATAA - 3′<br>Reverse 5′-ATTATGAGCTCTGAAGTCAAGAAAAGGGTGG - 3′
3	LRPPRC	Forward 5′-ATTATACTAGTAATAACCAGGCGATACTTTG - 3′<br>Reverse 5′-ATTATAAGCTTATAGGGATCACTTTTATTTC - 3′
4	DRG1	Forward 5′-ATTATACTAGTAACCTTTCCCTTTTCCCATC - 3′<br>Reverse 5′-ATTATAAGCTTTGCATGTAGCTGACCAGCCT - 3′
5	LYN	Forward 5′-ATTATACTAGTAGCACAGGGAGACCCGTCCA - 3′<br>Reverse 5′-ATTATAAGCTTTAAACAAGTAAGCCAAAGCA - 3′
6	SFPQ	Forward 5′-ATTATACTAGTATGTGATATTTAGGCTTTCA - 3′<br>Reverse 5′-ATTATGAGCTCCATGTTTAACATCTTTAATT - 3′
7	ALDH5A1	Forward 5′-ATTATACTAGTTATCCTTGATCAATTTTCTA - 3′<br>Reverse 5′-ATTATAAGCTTTACAGGGAGAGGAAATTCTG - 3′
8	GMFB	Forward 5′-ATTATACTAGTGGTAATCACAGTATTATTTA - 3′<br>Reverse 5′-ATTATAAGCTTAATGTTAACATTTTATTTAA - 3′
9	SLC12A2	Forward 5′-ATTATACTAGTATGTTCTATACAGTGGACAG - 3′<br>Reverse 5′-ATTATAAGCTTCTTGAGTATTTTTGTTTAAA - 3′
10	AGPAT5	Forward 5′-ATTATACTAGTATCTGGTGGAAAGCTACAAT - 3′<br>Reverse 5′-ATTATAAGCTTTGAGATGCATATTTTCCTTT - 3′
11	DIDO1	Forward 5′-ATTATACTAGTACAGCTAGTGAAACCTTTCC - 3′<br>Reverse 5′-ATTATAAGCTTGTGAACAGCAAGCACTGAGG - 3′
